# Nutrition as preventive medicine: a call for integration into medical education

**DOI:** 10.3389/fpubh.2025.1653382

**Published:** 2025-09-11

**Authors:** Shirley Kalwaney, Elizabeth Cerceo

**Affiliations:** ^1^Graduate Medical Education, Falls Church, VA, United States; ^2^Inova Fairfax Medical Campus, Inova Health System Falls Church, Falls Church, VA, United States; ^3^Department of Medicine, Cooper Medical School of Rowan University, Cooper University Healthcare, Camden, NJ, United States

**Keywords:** plant-based diet, medical education, nutrition education, nutrition education and chronic disease, plant-forward diet

The recent announcement by Health Secretary Robert F. Kennedy Jr. that medical schools must offer nutrition courses or risk losing federal funding from the Department of Health and Human Services represents a watershed moment for American medical education (1). Kennedy's declaration that “there's almost no medical schools that have nutrition courses” and his commitment to withhold funding from institutions that fail to implement comprehensive nutrition programs signals an unprecedented opportunity to address a fundamental gap in physician training. This mandate builds upon the growing recognition of this educational deficit, recently highlighted by the 2022 bipartisan passage of House Resolution 1118 in the United States (U.S.) House of Representatives, which called for meaningful nutrition education for medical trainees (2). This mandate arrives at a critical juncture when diet-related chronic diseases continue to devastate American health, making nutrition education not merely beneficial but essential for the future of medical practice.

The current state of nutrition education in medical training is inadequate to meet the mounting health challenges facing our patients. In the most recent survey, medical students spend an average of only 19 h on required nutrition education over their entire 4-year curriculum failing to meet the even the minimum hours recommended 40 years ago (3). This limited exposure focuses predominantly on biochemistry and vitamin deficiency states -conditions that are not major health problems in the U.S. today. Following medical school, nutrition education during graduate medical education is minimal or, more commonly, entirely absent. The Accreditation Council for Graduate Medical Education's Common Program Requirements lack any requirement for physicians-in-training to learn about nutrition or diet, a glaring omission given the central role of nutrition in disease prevention and management (4).

This educational deficit becomes even more concerning when examined against the backdrop of America's chronic disease epidemic. Diet-related diseases contribute to over 11 million premature deaths annually worldwide, with poor-quality diet identified as the leading cause of death in the United States (5). More than two-thirds of American adults are overweight or obese, and half have diabetes or pre-diabetes, conditions that disproportionately affect vulnerable populations (6, 7). These statistics represent not just individual tragedies but a systemic failure to prioritize prevention through evidence-based nutritional interventions.

The evidence supporting nutrition as a therapeutic intervention is overwhelming and continues to grow. Randomized clinical trials have demonstrated that dietary interventions can both prevent and manage critical diseases, including diabetes and cardiovascular disease. Mediterranean-style diets have been shown to reduce recurrent cardiovascular events by 72%, representing an absolute difference of 2.83 events per year compared with control groups (7). For individuals with elevated fasting blood glucose, combining dietary changes with physical activity reduced the risk of developing diabetes by 58%—nearly double the 31% reduction achieved with metformin alone (8). These outcomes rival or exceed many pharmaceutical interventions, yet physicians receive minimal training in implementing such dietary strategies.

Current clinical care guidelines recognize nutrition as a primary intervention, with the American Heart Association and American College of Cardiology positioning “healthy lifestyle” at the apex of their treatment algorithms for both primary and secondary prevention of atherosclerotic cardiovascular disease (9). The disconnect between guideline recommendations and physician preparedness raises a fundamental question: how can clinicians effectively implement these evidence-based recommendations without adequate training in nutrition?

Five compelling reasons underscore why nutrition education deserves special attention in medical training reform. First, the U.S. Burden of Disease Collaborators have identified poor-quality diet as the leading cause of death in the United States, with the prevalence and cost of diet-related diseases predicted to continue climbing if left unchecked (10, 11). Second, there is renewed interest in shifting healthcare from disease management to health promotion and prevention- areas that physicians will find difficult to advance without a solid foundation in nutrition education and counseling (12). Third, patients are bombarded with confusing and often contradictory nutrition messages from media sources, creating an urgent need for physicians to provide authoritative, evidence-based guidance. Fourth, there is increasing attention on the wellness and self-care of residents and fellows, and nutrition education has the potential to enhance physician wellbeing while making them more effective counselors. Lastly, the economic case for nutrition education is becoming increasingly compelling as primary prevention makes more economic sense than expensive secondary and tertiary care.

Frameworks should encompass multiple educational strategies across different competency domains. Medical knowledge competencies should include understanding the relationship between diet quality and cardiometabolic health, while interpersonal and communication skills should encompass effective dietary counseling techniques that consider patient goals and social determinants of health. Clinicians who are going into fields such as family medicine, obstetrics, pediatrics, or internal medicine in particular should have targeted, clinically relevant nutrition education in residency training as it may not be feasible to cover detailed clinical information comprehensively within an already dense undergraduate medical curriculum. Interdisciplinary collaboration and communication with nutrition experts can support and enhance clinical decision-making and promote effective patient care. Practice-based learning and improvement competencies should address cost-effectiveness of dietary interventions and quality improvement projects targeting nutritional factors. Systems-based practice competencies should explore food accessibility, environmental determinants of health, and advocacy for policy changes that support healthy dietary patterns (Table 1).

**Table 1:** Competency framework for nutrition education and counseling.

**Competency domain**	**Educational objectives**	**Sample educational strategies**
Patient care	Elicit brief diet/food history; anthropometrics, targeted labs, screen for food/nutrition insecurity and deficiencies	Interprofessional workshops for “5-min nutrition” micro-visits; OSCE/SP on food insecurity & counseling
Medical knowledge	Understand the relationship between diet quality and cardiometabolic health; grasp foundational principles of nutrition science	Integrated lectures on nutrition and chronic disease states; case-based learning modules on dietary interventions
Interpersonal & communication skills	Develop skills to counsel patients on dietary changes, consider cultural preferences, and respect patient autonomy in dietary decisions	Simulated patient counseling sessions; role-playing scenarios using scripts; motivational interviewing workshops; start non-judgmental food conversations; use MI; co-create SMART goals; address bias; use interpreters effectively
Practice-based learning & improvement	Engage in continuous quality improvement related to nutrition interventions and analyze cost-effectiveness of dietary approaches	Quality improvement projects on nutrition screening or malnutrition; journal clubs evaluating nutrition-related interventions
Systems-based practice	Identify and address food access issues; advocate for policies supporting healthy eating environments; understand food systems' impact on health	Community rotations focusing on food access; policy advocacy exercises; environmental health seminars; hospital food-environment audits
Professionalism	Promote patient-centered dietary care with sensitivity to cultural, ethical, and social factors	Ethics discussions on nutrition and public health; reflective writing assignments on clinician dietary practices
Interprofessional collaboration	Collaborate effectively with dietitians, nurses, and allied health professionals to deliver cohesive nutritional care	Team-based learning exercises; interprofessional simulations (RD, RN, SW); joint care planning with allied health professionals

The integration of comprehensive nutrition education into medical training demands that nutrition should not be considered an isolated discipline but rather a vital, practical determinant of health woven throughout existing curricula. Beyond curricular changes, healthcare institutions can demonstrate their commitment to nutrition by aligning their practices with their educational messages. Medical training programs and conferences should serve foods that align with the USDA Dietary Guidelines for Americans and AHA Healthy 8, replacing the highly and ultra-processed foods commonly found at medical gatherings (9).

Some might argue that physicians do not need extensive nutrition education because other healthcare professionals, including dietitians, are better positioned to make dietary recommendations. However, when physicians understand and adopt healthy lifestyle behaviors, they are often more effective at guiding their own patients toward healthy changes (13). Organizations such as the American College of Lifestyle Medicine which certifies healthcare professionals in lifestyle medicine, including nutrition have successfully included nutrition as a core component of medical education, along with specific strategies these institutions have adopted, would enrich the discussion and demonstrate how such efforts are shaping the future of medical training. In addition, healthy behavior change requires a coordinated team effort that can include appropriately trained dietitians, nutritionists, nurses, health coaches, and culinary professionals. The problem is that most physicians currently lack sufficient education to contribute meaningfully to this team. At minimum, physicians need adequate training to initiate nutrition conversations with patients and make effective referrals and address behavior change during patient encounters (14).

Moreover, a more balanced and impactful approach to patient care recognizes the value of interdisciplinary care management. A well-trained healthcare team consisting of nurse practitioners, physician assistants, dietitians, community health workers, and other allied health professionals can drive improvements in chronic disease management, reduce healthcare costs, and enhance outcomes across diverse populations. This collaborative model is especially critical in rural and underserved communities where physician shortages persist. In such settings, empowering the broader care team with shared nutritional knowledge ensures that patients receive consistent, evidence-based dietary guidance. Integrating nutrition competencies across disciplines also enhances team-based care, promoting shared decision-making, continuity, and patient trust.

Secretary Kennedy's directive represents a historic opportunity to transform medical education and improve patient outcomes through evidence-based nutrition training. The medical community must embrace this moment to build comprehensive, integrated nutrition curricula that prepare physicians to address the chronic disease epidemic. By equipping future physicians with the knowledge and skills to promote nutritious dietary patterns, we can achieve better health outcomes for our patients while reducing healthcare costs and advancing the fundamental mission of medicine: to prevent disease and promote health (15). In an era marked by escalating diet-related disease burdens, incremental progress is no longer sufficient. A comprehensive, interdisciplinary approach to nutrition education must now be recognized as an essential component of patient care and public health.
